# Of Cross-immunity, Herd Immunity and Country-specific Plans: Experiences from COVID-19 in India

**DOI:** 10.14336/AD.2020.1104

**Published:** 2020-12-01

**Authors:** Sankha Shubhra Chakrabarti, Upinder Kaur, Anup Singh, Suddhachitta Chakrabarti, Manigreeva Krishnatreya, Bimal Kumar Agrawal, Amit Mittal, Amit Singh, Rahul Khanna, Indrajeet Singh Gambhir, Kunlin Jin, Sasanka Chakrabarti

**Affiliations:** ^1^Department of Geriatric Medicine, Institute of Medical Sciences, Banaras Hindu University, Varanasi, India.; ^2^Department of Pharmacology, Institute of Medical Sciences, Banaras Hindu University, Varanasi, India.; ^3^Department of Geriatric Medicine, Institute of Medical Sciences, Banaras Hindu University, Varanasi, India.; ^4^Health Department, Kolkata Municipal Corporation, India.; ^5^Department of Cancer Registry and Epidemiology, Dr. B Borooah Cancer Institute, Guwahati, India.; ^6^Department of General Medicine, Maharishi Markandeshwar (deemed to be) University, Mullana, India.; ^7^Department of Radiodiagnosis, Maharishi Markandeshwar (deemed to be) University, Mullana, India.; ^8^Department of Pharmacology, Institute of Medical Sciences, Banaras Hindu University, Varanasi, India.; ^9^Department of General Surgery, Institute of Medical Sciences, Banaras Hindu University, Varanasi, India.; ^10^Department of Geriatric Medicine, Institute of Medical Sciences, Banaras Hindu University, Varanasi, India.; ^11^Department of Pharmacology and Neuroscience, University of North Texas Health Science Center, Fort Worth, Texas, USA.; ^12^Department of Biochemistry and Central Research Cell, Maharishi Markandeshwar (deemed to be) University, Mullana, India.

**Keywords:** lockdown, T cells, integration, hygiene hypothesis, restructuring

## Abstract

India has witnessed a high number of COVID-19 cases, but mortality has been quite low, and most cases have been asymptomatic or mild. In early April, we had hypothesized a low COVID-19 mortality in India, based on the concept of cross-immunity. The presence of cross-immunity is presumed to lead to a milder course of disease and allow the time necessary for the development of adaptive immunity by the body to eliminate the virus. Evidence supporting our hypothesis has started showing up. Multiple studies have shown the generation of different T cell subsets and B cells responding to epitopes of viral proteins, especially of the spike protein, as a part of adaptive immunity against SARS-CoV-2. Cross-reactive T-cells have been demonstrated in patients who have been previously exposed to endemic coronaviruses. The interplay of cross-immunity and herd immunity is apparent in the COVID-19 scenario in India from the presence of a large number of asymptomatic or mild cases, a low infection-fatality ratio and a generally flat curve of percentage positivity of cases with respect to total testing, both in periods of strict lock-down and step-wise unlocking. It seems that cross-immunity resulted in faster generation of herd immunity. Although the initial restrictive measures such as lockdown prevented the rapid spread of the outbreak, further extension of such measures and overly expensive ones such as enhanced testing in India will result in a huge burden on the health economics as well as the society. Hence, we propose a restructuring of the health services and approach to COVID-19. The restructured health services should move away from indiscriminate testing, isolation and quarantine, and instead, the emphasis should be on improving facilities for testing and management of only critical COVID cases and the replacement of complete lockdowns by the selective isolation and quarantine of susceptible persons such as the aged and those with co-morbidities. In the process of describing India-specific plans, we emphasize why the development of country-specific plans for tackling epidemics is important, instead of adopting a “one policy fits all” approach.

## 1. Introduction

In early April, we hypothesized that COVID-19 mortality would be lower in India by global standards, and the overall impact of COVID-19 in additive mortality may be low [1]. Over the past six months, COVID-19 cases have increased in India. In contrast to case numbers, however the mortality has consistently come down since mid-May, from a peak of 3.7% to as low as 1.5% (www.mohfw.gov.in/, http://health.delhigovt.nic.in/wps/wcm/connect/doit_health/Health/Home/Covid19/Bulletin+July+2020, https://ourworldindata.org/coronavirus). The trend visible even after India started opening up its cities, transport and other services since 1st June, suggests that the mortality ratio would come down further. This is possibly attributable to an increasing number of asymptomatic and mildly symptomatic COVID-19 patients who are getting identified due to increased testing rates. Serological surveys provide further evidence of a low infection mortality ratio in India. These have been conducted by accredited private laboratories in several Indian cities though without random sampling and based on on-demand testing. Such surveys have also been conducted by municipal authorities in many of the metropolitan cities in India on randomly sampled populations using Indian Council of Medical Research (ICMR) approved IgG ELISA kits which are deemed to be highly specific. Most of these survey findings have only made it to newspaper and electronic media reports and not to peer reviewed literature, but mostly they have claimed a positivity between 15-30% in asymptomatic healthy individuals. This implies that many Indians have already been infected with SARS-CoV-2, recovered and developed antibodies to the virus, without even being aware of such infection; the process resulting in probable herd immunity to severe disease (https://frontline.thehindu.com/the-nation/far-from-herdimmunity/article32303481.ece). A study published by the ICMR in late September 2020 on findings of country-wide sero-surveys conducted in the months of May and June 2020, revealed an infection fatality ratio of only 0.15%, that too in districts with presumed robust death reporting. This value seems quite insignificant compared to other major diseases prevalent in India [2]. Thus, the suggestion of some experts for further increase in testing rate by RT-PCR for COVID-19 in India appears superfluous. India, despite the low per capita testing, in absolute numbers stands second only to the US, a remarkable feat for a developing country with a doctor-people ratio of 0.9 per 1000, lower than even its south Asian neighbors (https://data.worldbank.org/indicator/%20SH.MED.PHYS.ZS?most_recent_value_desc=true). The testing rate in India has increased from a few thousands per day in March, 2020 to over 1 million per day in October, 2020.

**Figure 1.**
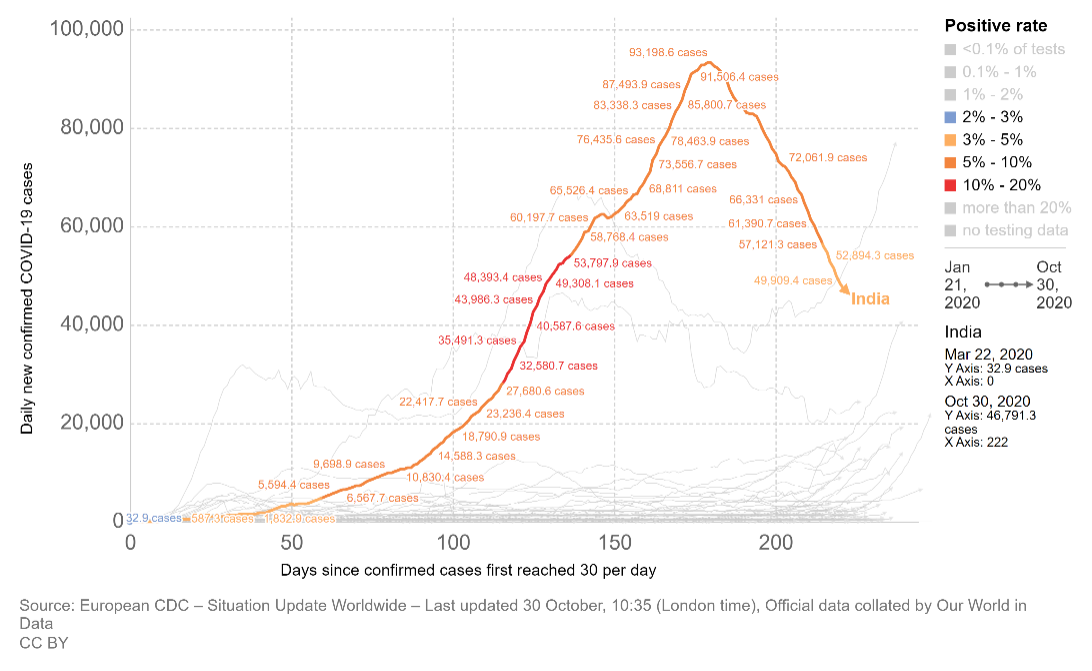
The trend of COVID-19 cases in India. X-axis shows days since confirmed cases first reached 30/day (22 March 2020). Y-axis shows daily new confirmed COVID-19 cases. There is a visible decline in daily number of cases from mid-September 2020 onwards. Lockdown restriction easing started since early June 2020. The color of the trendline represents the most important statistic of percentage positivity of tests conducted daily. For a major part between day-100 and day-150, the line color is red (>10% positivity). This has come down to <5% positivity now (line color light orange), implying a general decrease in SARS-CoV-2 infections despite lifting of most lockdown restrictions.

Interestingly, unlike other western nations who are also on the decline in mortality ratio, India has not had a noticeable peak in mortality ratio. Further, as per government records, and also in the authors’ experience, most patients dying with COVID-19 had significant co-morbidities like cardio-vascular dysfunction, diabetes, renal failure, and COPD. This clearly indicates that COVID-19 is predominantly fatal only for a vulnerable group in the Indian population rather than being a disease with high mortality (https://pib.gov.in/PressReleasePage.aspx?PRID=1628696). The suggestion by some groups that there has been under-reporting of COVID-19 deaths in India is hard to believe as RT-PCR testing for SARS-CoV-2 is widely available free of cost, and patients likely to die *i.e*. the critical cases are almost always being tested as per the authors’ cumulative experience of providing six months of COVID care in diverse settings.

Another interesting feature comes out when we calculate the percentage of positive cases in India with respect to number of tests performed daily. There has not been any remarkable peak in this statistic over time; the percentage positivity with respect to number of tests performed daily was around 3% in March, increased to more than 10% slowly but steadily both during the periods of strict lock-down and stepwise unlocking. The percentage positivity has now come down to below 5% (Fig. 1) (https://ourworldindata.org/coronavirus). This phenomenon clearly points to the presence of biological factors that are attenuating the spread of the COVID-19 outbreak in India, as was predicted in our April 2020 paper [1].

A lock-down was imposed early on by the Indian government and was managed proactively and efficiently by the Central and various State governments. This expectedly brought down the absolute number of positive cases and absolute number of COVID-19 deaths in the vulnerable groups within a defined period of time. It also allowed the state health machinery to gear up for better diagnostic and therapeutic management of COVID-19 patients with limited resources. The performance of India thus looks impressive in comparison to many advanced Western countries if one considers per million mortality. However, not only is the absolute mortality much lower than can be explained by proactive public health management, the huge number of asymptomatic or mildly symptomatic patients of COVID-19 in India as well as the slow and steady spread of the disease (testing positivity ratio) too are not explained without taking recourse to biological factors.

## 2. Cross-immunity and Herd immunity

In our April paper, based on our cumulative clinical experience of managing viral infections in the elderly, cancer patients, and diverse other groups in India, we had proposed that cross-immunity from exposure to other human coronaviruses (HCoVs) endemic in the population would be the major factor resulting in lower mortality and morbidity in Indians [1]. The four endemic coronaviruses- HCoV-229E, HCoV-NL63, HCoV-OC43, HCoV-HKU1 are responsible for respiratory illness which is usually self-limiting in immune-competent persons. We suggested that both neutralizing antibodies and primed T-cells generated because ofendemic coronavirus infections may be protective against COVID-19 [1]. Evidence has now started emerging in line with our hypothesis. One of the first evidence of cross-immunity came from the work of Grifoni and others. The authors quantitated SARS-CoV-2 specific CD4+ T-cell responses in patients who had recovered from lab-confirmed COVID-19, as well as healthy controls whose samples had been collected between 2015-2018, excluding any possibility of SARS-CoV-2 infection. Expectedly, spike and non-spike specific CD4+ T-cell responses were observed in the recovered patients but interestingly, non-spike-specific CD4+ T-cell responses were also positive in 50% of unexposed serum donors. These donors were found to have IgG antibodies against the endemic coronaviruses HCoV-OC43 and HCoV-NL63 [3]. Subsequently, similar findings have been reported from the Netherlands, Germany, Singapore and the UK [4]. This is also corroborated by a recent study published in Science by Sette and others. 142 T-cell epitopes were mapped across the SARS-CoV-2 genome (66 in the Spike protein region and 76 from the rest of the genome) to examine CD4+ T cell response to these. CD4+ T memory cells from sero-negative SARS-CoV-2 unexposed subjects cross-reacted with SARS-CoV-2 and also common HCoVs with respect to epitopes in Spike (S), nsp8, nsp12, nsp13 and N proteins. It was further observed in this study that SARS-CoV-2 unexposed subjects were sero-positive for antibodies against HCoV-NL63, HCoV-HKU1 and HCoV-OC43 [5]. So far there is no clear evidence of circulating anti-bodies to common HCoVs cross-reacting with SARS-CoV-2. However, multiple studies have demonstrated the existence of neutralizing antibodies and memory B cells recognizing different epitopes of SARS-CoV-2 proteins, especially of the S protein, in the peripheral circulation of COVID-19 convalescent patients [6-8]. Some of these SARS-CoV-2 specific memory B cells and serum IgG are also cross-reactive with the endemic coronavirus HCoV-OC43 [9].

Previously cross-reactive antibodies against HCoV-229E and HCoV-OC43 have been demonstrated in those infected by SARS-CoV [10]. Similarly, SARS-CoV patients in convalescence have been shown to contain neutralizing antibodies against MERS-CoV; and sera of convalescent patients of SARS-CoV contain neutralizing antibodies that inhibit the entry of SARS-CoV-2 in Vero cells [11,12]. Thus, it appears that the generation of cross-reactive antibodies across many different types of coronaviruses following infection by one particular HCoV is a common phenomenon. We suggest that such neutralizing antibodies together with memory T and B cells against common HCoVs may confer cross-immunity against SARS-CoV-2.

The situation in India needs to be analyzed in the backdrop of emerging evidence of cross-immunity. The cross-immunity will result in asymptomatic or milder course of COVID-19 in many people and it will allow time for the body to launch suitable adaptive immune responses for viral elimination. Attainment of herd immunity seems easier in countries such as India due to another reason too. Unidentified viral infections are rampant in India, to a large extent attributable to generally poor standards of hygiene, and lack of widespread testing facilities. The national Integrated Disease Surveillance Programme has suggested that more than 70% outbreaks of infectious diseases in the country are due to viruses [13]. Under such a continuous onslaught of viral infections- often multiple times in the same individual in a year, cross-immunity and subsequently herd immunity may be easier to acquire in India. The epidemiologic evidence of COVID-19 in India so far supports this. Percentage positivity for SARS-CoV-2 with respect to total number of daily PCR tests and the case mortality ratio have both decreased even after significant easing of lockdown restrictions.

## 3. A COVID plan specific to India

This brings us to the question of designing nation-specific strategies instead of trying to make every country fit one design of testing, contact tracing, quarantining, and lockdown. In less immune populations with strong health resources, this strategy of quarantining the infected to protect others may be successful. In contrast, in nations with low mortality and a predominantly immune population, it would be more logical to protect the vulnerable subjects and limit restrictive policies such as total lockdowns. Over the past six months, almost the entire Indian administrative machinery and health system has been focused on tackling COVID-19. While the governments, both the central and states have done a commendable job to provide care to these patients and to ensure the steady supply of necessary goods, the cost-benefit ratio needs to be carefully measured. A total lock-down for a short period limits the spread of an outbreak and prevents large number of deaths among vulnerable subjects in a short span of time, thus allowing the health care system ample time to gear up for a challenge. Following this brief lockdown, restrictive measures should only be applied to vulnerable subjects and in the COVID-19 context it includes the elderly and those with co-morbidities like diabetes, cardio-vascular diseases, chronic kidney diseases, immune-suppression *etc*.

In India because of a prolonged lock-down, migrating labor forces have been out of work, other jobs have been lost and domestic tourism has suffered. School-going children in rural areas without access to quality online modes of education have faced disparity in learning. Non-COVID healthcare too has taken a hit due to travel restrictions, absence of public transport, and shunting of already sparse healthcare machinery towards a disease which does not match up to any of India’s priority health issues such as tuberculosis, anemia, malnutrition, diarrheal diseases or even chronic diseases of lifestyle in terms of mortality. The elderly who are an already neglected part of the Indian demographic have suffered more severely as family members have been unable to deal with the continuous media campaign of fear and elderly vulnerability to COVID-19. In the authors’ own experience, several older follow-up patients especially from the rural areas have died due to not being brought to the hospital for non-COVID emergency health needs. The absence of public transport has also made it difficult for the elderly to be brought to urban centers for healthcare. Another group which has suffered are patients with cancer. Many of them who could not afford private healthcare facilities due to financial constraints might have succumbed at home. Indirect evidence for this comes from a significant decline of up to 50% in new cancer patient registrations in specialized cancer centers [14]. It is also possible that many such patients who could have been diagnosed at early stages would present for the first time in advanced stages, post-lockdown. Psychological stress and suicides have also increased, including among the medical fraternity (https://indianexpress.com/article/cities/delhi/another-aiims-doctor-commits-suicide-third-in-the-past-month-6555338/) [15,16]. It is worth remembering that India does not rank high up with western democracies in per capita GDP, or in health expenditure and health manpower. However, India did adopt the western strategy of testing more and more patients, even those with relatively less or no symptoms. Early on this may have been logical considering that much was unknown about COVID-19 but now it seems counter-productive as evidence of low mortality and high immunity in India reveals itself. An interesting snippet concerns the world’s largest slum Dharavi in Mumbai- the commercial capital of India. On the 11^th^ of July, the WHO praised implementation of this very model in the densely populated Dharavi slum in Mumbai, stating “testing, massive screening, identifying patients and their high risk contacts, and isolating them from the community has worked very well for Dharavi” (www.hindustantimes.com/mumbai-news/who-praises-dharavi-s-covid-fight/story-quFz7j1kyguZztFCwN8ZtK.html). However, a study in late July by the Tata Institute of Fundamental Research demonstrated a 57% seropositivity for SARS-CoV-2 antibodies in the population screened from Mumbai slums, implying that flattening of the curve of COVID-19 cases in these areas could be ascribed more correctly to development of herd immunity rather than the testing-isolation-quarantine strategy (www.livemint.com/news/india/mumbai-sero-prevalence-of-57-found-in-slums-and-16-in-residential-societies-11595952896909.html). Testing-isolation-quarantine seems neither practical nor implementable in a country like India, especially for prolonged periods. In the authors’ opinion, an ideal Indian testing strategy should be focused on severe COVID-19 cases, symptomatic patients with major comorbidities or those patients undergoing invasive medical or surgical procedures. We understand that it would be very difficult for the Government to convince the Indian population in general of the benefits of lower testing, the public having been continuously bombarded by the media with western norms of increased testing. However, restricting expenditure on RT- PCR tests would allow the government to divert funds to other priority health areas which are missing out. Further, a prudent approach in India would be to protect the elderly and those with major co-morbidities and provide faster healthcare to these vulnerable groups, rather than trying to segregate younger healthy patients with no or minimal symptoms. COVID-19 management centers should also focus on serious cases with ARDS or other complications. Lateral integration of COVID-19 care with non-COVID services is of utmost importance, so that comorbidities may be adequately tackled. Vaccine development and disbursal should also be focused on vulnerable groups. Although not entirely relevant in the present discussion, we suggest that drug repurposing and trials on low cost medications such as hydroxychloroquine, ivermectin, bromhexine, ammonium chloride, angiotensin converting enzyme inhibitors, and anticoagulants must be priority research areas, rather than fixation on drugs such as remdesivir and tocilizumab which create major financial burden on the already stretched health resources of the country [17,18].

## 4. Conclusion

In a country which seems to be naturally protected against severe manifestations of COVID-19, a restructuring of the health policy is needed to bring the economy, social life, as well as healthcare back on track. Otherwise, the financial and non-COVID health implications of current strategies may be too severe for India to recover from, in the long run. The Indian experience also emphasizes the need for developing tailored policies for epidemic handling based on the epidemic-characteristics in the concerned region and biological factors prevalent there, rather than trying to follow ‘One strategy fits all’.

## References

[1] Chakrabarti SS, Kaur U, Banerjee A, Ganguly U, Banerjee T, Saha S, et al (2020). COVID-19 in India: Are Biological and Environmental Factors Helping to Stem the Incidence and Severity? Aging Dis, 11(3):480. 32489695 10.14336/AD.2020.0402 PMC7220291

[2] Murhekar M, Bhatnagar T, Selvaraju S, Rade K, Saravanakumar V, Vivian Thangaraj J, et al (2020). Prevalence of SARS-CoV-2 infection in India: Findings from the national serosurvey, May-June 2020. Indian J Med Res, 152(1):48. 32952144 10.4103/ijmr.IJMR_3290_20 PMC7853249

[3] Grifoni A, Weiskopf D, Ramirez SI, Mateus J, Dan JM, Moderbacher CR, et al (2020). Targets of T Cell Responses to SARS-CoV-2 Coronavirus in Humans with COVID-19 Disease and Unexposed Individuals. Cell, 181(7):1489-1501.e15. 32473127 10.1016/j.cell.2020.05.015 PMC7237901

[4] Sette A, Crotty S (2020). Pre-existing immunity to SARS-CoV-2: the knowns and unknowns. Nat Rev Immunol, 20(8):457-8. 32636479 10.1038/s41577-020-0389-z PMC7339790

[5] Mateus J, Grifoni A, Tarke A, Sidney J, Ramirez SI, Dan JM, et al (2020). Selective and cross-reactive SARS-CoV-2 T cell epitopes in unexposed humans. Science, 370:89-94. 32753554 10.1126/science.abd3871 PMC7574914

[6] Rogers TF, Zhao F, Huang D, Beutler N, Burns A, He W, et al (2020). Isolation of potent SARS-CoV-2 neutralizing antibodies and protection from disease in a small animal model. Science, 369:956-63. 32540903 10.1126/science.abc7520 PMC7299280

[7] Robbiani DF, Gaebler C, Muecksch F, Lorenzi JCC, Wang Z, Cho A, et al (2020). Convergent antibody responses to SARS-CoV-2 in convalescent individuals. Nature, 584(7821):437-42. 32555388 10.1038/s41586-020-2456-9 PMC7442695

[8] Liu L, Wang P, Nair MS, Yu J, Rapp M, Wang Q, et al (2020). Potent neutralizing antibodies against multiple epitopes on SARS-CoV-2 spike. Nature, 584(7821):450-6. 32698192 10.1038/s41586-020-2571-7

[9] Nguyen-Contant P, Embong AK, Kanagaiah P, Chaves FA, Yang H, Branche AR, et al (2020). S Protein-Reactive IgG and Memory B Cell Production after Human SARS-CoV-2 Infection Includes Broad Reactivity to the S2 Subunit. Ellebedy A, Schultz-Cherry S, editors. MBio, 11(5). 10.1128/mBio.01991-20 PMC7520599 32978311

[10] Che X, Qiu L, Liao Z, Wang Y, Wen K, Pan Y, et al (2005). Antigenic Cross-Reactivity between Severe Acute Respiratory Syndrome-Associated Coronavirus and Human Coronaviruses 229E and OC43. J Infect Dis, 191(12):2033-7. 15897988 10.1086/430355 PMC7109809

[11] Chan K-H, Chan JF-W, Tse H, Chen H, Lau CC-Y, Cai J-P, et al (2013). Cross-reactive antibodies in convalescent SARS patients’ sera against the emerging novel human coronavirus EMC (2012) by both immunofluorescent and neutralizing antibody tests. J Infect, 67(2):130-40. 23583636 10.1016/j.jinf.2013.03.015 PMC7112694

[12] Hoffmann M, Kleine-Weber H, Schroeder S, Krüger N, Herrler T, Erichsen S, et al (2020). SARS-CoV-2 Cell Entry Depends on ACE2 and TMPRSS2 and Is Blocked by a Clinically Proven Protease Inhibitor. Cell, 181(2):271-280.e8. 32142651 10.1016/j.cell.2020.02.052 PMC7102627

[13] Mourya DT, Yadav PD, Ullas PT, Bhardwaj SD, Sahay RR, Chadha MS, et al (2019). Emerging/re-emerging viral diseases & new viruses on the Indian horizon. Indian J Med Res, 149(4):447-67. 31411169 10.4103/ijmr.IJMR_1239_18 PMC6676836

[14] Kataki A, Krishnatreya M (2020). Cancer care and COVID-19 pandemic: An experience from cancer center in North-East India. Curr Med Issues, 18(3):213.

[15] Dsouza DD, Quadros S, Hyderabadwala ZJ, Mamun MA (2020). Aggregated COVID-19 suicide incidences in India: Fear of COVID-19 infection is the prominent causative factor. Psychiatry Res, 290:113145. 32544650 10.1016/j.psychres.2020.113145 PMC7832713

[16] Choudhari R (2020). COVID 19 pandemic: Mental health challenges of internal migrant workers of India. Asian J Psychiatr, 54:102254. 32593122 10.1016/j.ajp.2020.102254 PMC7301775

[17] Kaur U, Chakrabarti SS, Ojha B, Pathak BK, Singh A, Saso L, et al (2020). Targeting host cell proteases to prevent SARS-CoV-2 invasion. Curr Drug Targets, 21. 10.2174/1389450121666200924113243 32972339

[18] Kaur U, Acharya K, Mondal R, Singh A, Saso L, Chakrabarti S, et al (2020). Should ACE2 be given a chance in COVID-19 therapeutics: A semi-systematic review of strategies enhancing ACE2. Eur J Pharmacol, 887:173545. 32926917 10.1016/j.ejphar.2020.173545 PMC7485553

